# BRD9 Inhibition by Natural Polyphenols Targets DNA Damage/Repair and Apoptosis in Human Colon Cancer Cells

**DOI:** 10.3390/nu14204317

**Published:** 2022-10-15

**Authors:** Sabeeta Kapoor, Elisabetta Damiani, Shan Wang, Ravirajan Dharmanand, Chakrapani Tripathi, Jorge Enrique Tovar Perez, Wan Mohaiza Dashwood, Praveen Rajendran, Roderick Hugh Dashwood

**Affiliations:** 1 Center for Epigenetics & Disease Prevention, Texas A&M Health, Houston, TX 77030, USA; 2 Department of Life and Environmental Sciences, Università Politecnica delle Marche, 60121 Ancona, Italy; 3 Stomatology Hospital, School of Stomatology, Zhejiang University School of Medicine, Hangzhou 310030, China; 4 Center for Infectious & Inflammatory Diseases, Texas A&M Health, Houston, TX 77030, USA; 5 Department of Translational Medical Sciences, Texas A&M College of Medicine, Houston, TX 77030, USA

**Keywords:** apoptosis, bromodomain inhibition, colorectal cancer, molecular docking

## Abstract

Epigenetic mechanisms play an important role in the etiology of colorectal cancer (CRC) and other malignancies due, in part, to deregulated bromodomain (BRD) functions. Inhibitors of the bromodomain and extraterminal (BET) family have entered into clinical trials as anticancer agents, and interest has grown in other acetyl ‘reader’ proteins as therapeutic targets, including non-BET member bromodomain-containing protein 9 (BRD9). We report here that overexpression of BRD9 is associated with poor prognosis in CRC patients, and that siRNA-mediated knockdown of BRD9 decreased cell viability and activated apoptosis in human colon cancer cells, coincident with increased DNA damage. Seeking natural compounds as BRD9 antagonists, molecular docking in silico identified several polyphenols such as Epigallocatechin-3-gallate (EGCG), Equol, Quercetin, and Aspalathin, with favorable binding energies, supported by BROMOscan^®^ (DiscoverX) and isothermal titration calorimetry experiments. Polyphenols mimicked BRD9 knockdown and iBRD9 treatment in reducing colon cancer cell viability, inhibiting colony formation, and enhancing DNA damage and apoptosis. Normal colonic epithelial cells were unaffected, signifying cancer-specific effects. These findings suggest that natural polyphenols recognize and target BRD9 for inhibition, and might serve as useful lead compounds for bromodomain therapeutics in the clinical setting.

## 1. Introduction

CRC is a leading cause of cancer-related death worldwide, characterized by the accumulation of genetic and epigenetic alterations, including deregulated bromodomain (BRD) functions [1,2,3]. BRDs are evolutionarily conserved epigenetic ‘reader’ modules that recognize acetylated lysine residues on target proteins [4,5]. BRD-containing factors link acetylated histone and nonhistone proteins to the activation of RNA polymerase II, with critical roles in gene transcription, splicing, chromatin remodeling, protein scaffolding, and signal transduction [6]. Bromodomain-containing protein 9 (BRD9) is a recently identified subunit of the ATP-dependent chromatin remodeling switch/sucrose non-fermentable (SWI/SNF) complex that interacts with chromatin partners and transcription factors implicated in cell proliferation, apoptosis, differentiation, and cancer [7,8]. BRD9 also is involved in DNA replication, DNA repair, and cellular responses to DNA damage [9]; thus, targeting BRD9 provides opportunities for new avenues in cancer treatment. Bromodomain and extraterminal (BET) antagonists, such as JQ1, exhibit anticancer efficacy in clinical trials, but not without associated toxicity and drug resistance [10]. These observations have given impetus to the targeting of other BRD-containing proteins with a view to improved safety and efficacy. Nutrients from diet have an important role in the development of CRC, and more research is needed on specific foods and dietary compounds that can combat the development and progression of CRC. Dietary phytochemicals have been studied as modulators of epigenetic readers/writers/erasers [3,11,12], and the metabolites are untapped sources of novel bioactives for ‘drugging’ chromatin [3,13]. This report focuses on natural polyphenols as BRD9 inhibitors in CRC cells and the use of nutrients in targeting an epigenetic ‘reader’ protein deregulated in CRC.

## 2. Materials and Methods

### 2.1. Cells and Treatments

Human colon cancer cells (SW620, SW480) and non-transformed colonic epithelial cells (CCD841) were purchased from ATCC and used within 10–15 passages. Each cell line was confirmed independently to be of human origin, with no mammalian interspecies contamination, and with the correct genetic profile based on allele-specific markers (Idexx Radil, Columbia, MO, USA). Cells were cultured in McCoy’s 5A media (Invitrogen) or EMEM (Invitrogen), supplemented with 10% FBS and 1% penicillin/streptomycin, at 37 °C in a humidified chamber with 5% CO2. Chemicals, reagents, and cell culture media were from reputable commercial vendors, including supplies for pooled siRNAs, as reported [14,15,16].

### 2.2. Molecular Docking

Iterative docking of the BRD9 bromodomain with energy-minimized ligand was performed using reported methodologies [13,14,15,16,17], including SWISS-MODEL, AutoDock Vina, PDBePISA and LPC/CSU [18,19,20,21,22].

### 2.3. BROMOscan^®^ Screening

BRD9 inhibitor screening was performed using the BROMOscan^®^ Technology Platform (DiscoverX, Eurofins, Dallas, TX, USA) [23], with iBRD9 as a positive control and all compounds at a 10 µM final concentration. Test agents were blinded at the time of the assay.

### 2.4. Isothermal Titration Calorimetry

Experiments were conducted in a microcalorimeter (MicroCal PEAQ-ITC, Malvern Panalytical) using normal and reverse titration methods. The BRD9 protein was expressed in chemically competent *Escherichia coli* cells and purified by affinity chromatography. A microsyringe was loaded with 40-μL protein sample in isothermal titration calorimetry (ITC) buffer, in the concentration range 150−300 μM, and inserted into the calorimetric cell (0.2 mL cell volume), which was filled with test compound (10−30 μM) or protein (15−50 μM). Titrations involved 20–30 repeated 2-μL injections, with a duration of 4-s per injection. Titration experiments were optimized to ensure complete saturation of the protein or ligand before the final injection. Data were corrected by subtracting the heat of dilution, determined from independent titrations. MicroCal Origin 7.0 software was used to determine enthalpies of binding (ΔH) and binding constants (Kb), as reported [24].

### 2.5. Cell Viability

Cell Counting Kit-8 (CCK8, APExBIO, Boston, MA, USA) was used as reported [25]. Cells in the exponential growth phase were plated at a cell density of 5000 cells per well in 96-well tissue culture plates. After attachment overnight, cells were treated with compounds for 72 h across a broad range of concentrations, as shown in the figures. The colony formation assay also was performed, as described [26]. Cells were trypsinized and plated in 6-well dishes (500 cells/well), allowed to attach overnight, and then treated with test agents for 72 h. Seven days later, cells were fixed and stained with crystal violet. Colonies were counted and the surviving fraction was calculated as the ratio of the number of colonies in the treated sample to the number of colonies in the untreated control. Triplicate wells were established for each condition.

### 2.6. Immunoblotting

As reported [25], whole cell lysates (20 µg protein/lane) were separated by SDS-PAGE on 4–12% Bis-Tris gel (NuPAGE, Invitrogen, CA, USA) and transferred to nitrocellulose membranes (Invitrogen, CA, USA). Membranes were saturated with 2% Bovine Serum Albumin for 1 h, followed by overnight incubation at 4 °C with primary antibodies for BRD9 (1:1000, Active Motif, Carlsbad, CA, USA, #61537), pRPA32 S4/S8 (1:500, Bethyl Laboratories, Montgomery, TX, USA, #A300-245A), β-Actin (1:5000, Sigma-Aldrich, St. Louis, MO, USA, #A5441), c-Myc (1:1000, #D3N8F), pH2AX (1:500, #2577S), poly(ADP-ribose)polymerase (PARP) (1:1000, #9542), and cleaved caspase-3 (1:1000, #9661) from Cell Signaling (Danvers, MA, USA). After washing, membranes were incubated with horseradish peroxidase-conjugated secondary antibodies for 1 h. Bands were visualized using Western Lightning Plus-ECL Enhanced Chemiluminescence Substrate (Perkin Elmer, Waltham, MA, USA) and detected using a ChemiDoc MP Imaging System (Bio-Rad, Hercules, CA, USA).

### 2.7. Apoptosis

Colon cancer cells were treated with test compounds for 72 h in 6-well plates, and apoptosis assays were conducted as reported [27] using a BD Pharmingen PE Annexin V Apoptosis Detection Kit I (BD Biosciences, San Jose, CA, USA). Briefly, cells were collected, washed with PBS, and incubated in binding buffer with 5 μL of PE Annexin V for 5 min and 5 μL of 7-AAD for 15 min in the dark at 37 °C. Percent of apoptotic cells was determined using LSR II Flow cytometer (BD Biosciences) and FlowJo 10.8.1 software.

### 2.8. Statistics

Unless stated otherwise, findings are representative outcomes from three or more biological and technical replicates. Paired comparisons were made between test agent and vehicle control using Student’s *t*-test in GraphPad Prism 9.4.

## 3. Results

### 3.1. BRD9 Overexpression Is Associated with Reduced Survival in CRC Patients

**Figure 1:**
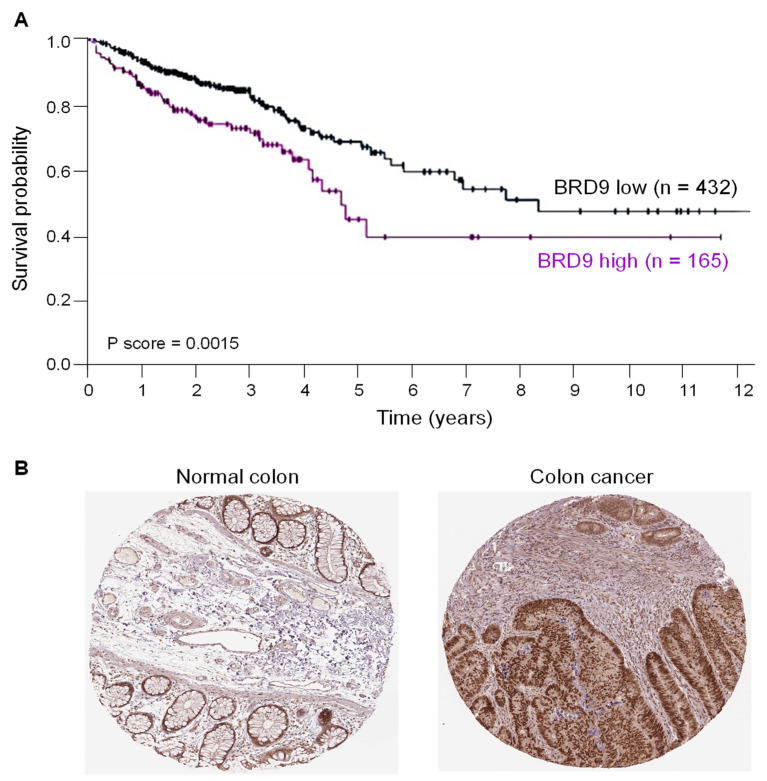
BRD9 overexpression is associated with reduced survival in CRC patients. (**A**) Overall survival probability in colorectal cancer (CRC) patients with high vs. low BRD9 mRNA expression in tumors (Human Protein Atlas at https://www.proteinatlas.org/ENSG00000028310-BRD9/pathology/colorectal+cancer, accessed on 13 June 2022). (**B**) Immunohistochemistry images of BRD9 protein expression in normal colon (https://www.proteinatlas.org/ENSG00000028310-BRD9/tissue/colon#img, accessed on 13 June 2022) and colon cancer (https://www.proteinatlas.org/ENSG00000028310-BRD9/pathology/colorectal+cancer#img, accessed on 13 June 2022).

Publicly available data revealed that Survival Probability was significantly worse in CRC cancer patients for whom BRD9 transcript expression was high in adenocarcinomas as compared with those harboring relatively low BRD9 levels (Figure 1A). In tissue microarrays, BRD9 protein was immunolocalized mainly to the nucleus, and was strongly positive in colon cancers as compared to normal colon (Figure 1B). These observations implicated an oncogenic role in CRC, and efforts to inhibit or downregulate BRD9.

### 3.2. BRD9 Regulates DNA Damage/Repair and Apoptosis in Human Colon Cancer Cells

**Figure 2:**
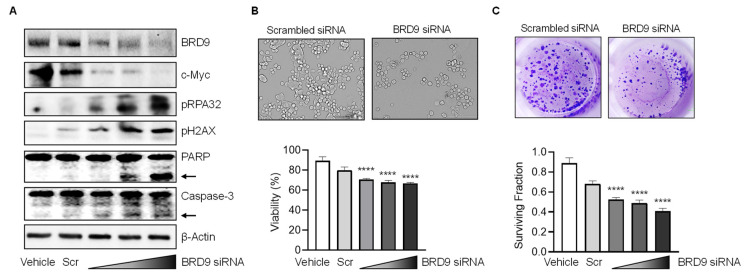
BRD9 depletion alters cell viability, DNA damage and apoptosis in human colon cancer cells. (**A**) Immunoblotting of SW620 cells 72 h after vehicle, scrambled (Scr) or BRD9 siRNA treatment. Knockdown experiments employed a pool of two different hairpin loop siRNAs; wedge symbol = 18.7, 37.5, 75 pmol siRNA transfected. β-Actin, loading control; arrows, positions of cleaved PARP and Caspase-3. (**B**) Representative morphology of SW620 cells and quantification of cell viability in the CCK-8 assay at 48 h. (**C**) Representative colony formation images and quantification of surviving fraction at 48 h. Data = mean ± SD, *n* = 3, ****p < 0.0001, by Student’s *t*-test vs. vehicle control.

In metastasis-lineage SW620 cells, transient transfection of BRD9 siRNAs for 72 h followed by immunoblotting revealed concentration-dependent reduction of BRD9 and c-Myc protein compared with vehicle-treated and scrambled siRNA-treated controls (Figure 2A). A concomitant increase was observed in pH2AX and pRPA32, which are well-characterized readouts of DNA damage and repair [16,25,28], along with cleaved PARP and caspase-3 apoptosis markers (Figure 2A, arrows). Morphological examination and quantification in the CCK8 assay revealed that siRNA-mediated BRD9 knockdown reduced cell viability significantly (Figure 2B), and there was a concentration-dependent decrease in the colony formation assay (Figure 2C). These findings indicated that BRD9 regulates DNA damage/repair, cell viability, and apoptosis induction in human colon cancer cells.

### 3.3. BRD9 Interacts with Natural Polyphenols

**Figure 3:**
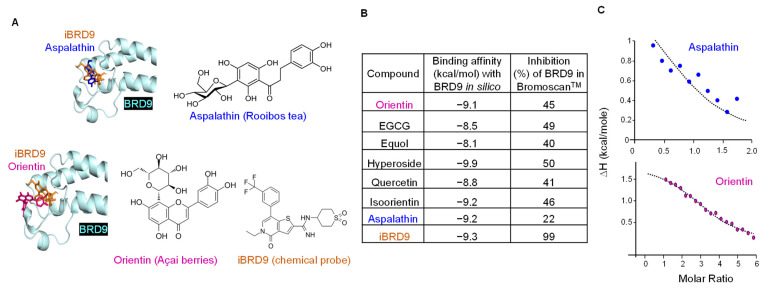
Interaction of natural polyphenols with BRD9. (**A**) Chemical structures of test agents and representative docking orientations in silico. (**B**) Binding affinities in silico and percent inhibition of BRD9 in the Bromoscan™ assay, with iBRD9 as a blinded positive control and 10 µM final concentration of each test agent. EGCG (Epigallocatechin-3-gallate), iBRD9 (Inhibitior of Bromodomain-containing protein 9) (**C**) Isothermal titration calorimetry (ITC) data for purified BRD9 protein with Aspalathin (Kd 20 µM, molar ratio N = 1) and Orientin (Kd 20 µM, molar ratio N = 3).

Extending prior molecular docking in silico [13,14,16], interactions of BRD9 were examined with various natural polyphenols (Figure 3A,B). Test agents bound to the acetyl lysine binding site with favorable orientations and binding energies in the range −8.1 to −9.9 kcal/mol, comparable to the synthetic chemical probe iBRD9 [29]. However, whereas 10 μM iBRD9 exhibited 99% inhibition in BROMOscan^®^ screens, agents such as Orientin and Aspalathin at the same concentration had 45% and 22% inhibitory activity, respectively (Figure 3B, right column). Additional positive controls corroborated the selectivity and specificity of BROMOscan^®^ screens, including JQ1 towards BRD4 and BI7273 for BRD7 (Supplementary Table S1). In ITC experiments, an apparent Kd value of 20 μM supported moderate reversible interactions with purified BRD9 protein, and predicted 1:1 or 3:1 molar ratios for Aspalathin and Orientin, respectively (Figure 3C).

### 3.4. BRD9 Inhibition Reduces Colon Cancer Cell Viability and Increases DNA Damage and Apoptosis

**Figure 4:**
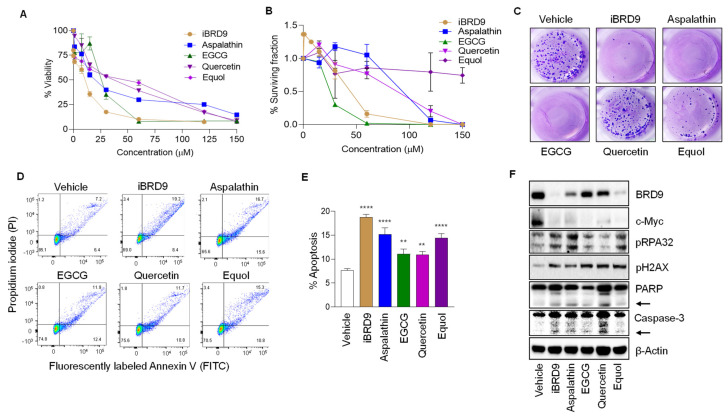
Polyphenols recapitulate BRD9 inhibitor phenotypes in colon cancer cells. (**A**) SW620 cell viability data from CCK-8 assay at 72 h. (**B**) Colony formation data showing surviving fraction (%). (**C**) Representative images of crystal violet-stained colonies after treatment with test compounds. (**D**) Apoptosis as detected by flow cytometry after treatment with 30 µM test compounds and (**E**) quantification of three biological replicates. Data = mean ± SD, *n* = 3. ** *p* < 0.01, **** *p* < 0.0001 by Student’s *t*-test vs. vehicle. (**F**) Immunoblotting of SW620 cell lysates after treatment with 30 µM of compounds. β-Actin, loading control; arrows, cleaved PARP and Caspase-3.

In metastasis lineage SW620 colon cancer cells, iBRD9 and predicted BRD9-inhibitory natural polyphenols exhibited concentration-dependent inhibition of cell viability and colony formation (Figure 4A–C), with a significant increase in the percentage of cells undergoing apoptosis (Figure 4D,E). The non-metastatic cell line SW480 obtained from the same patient as SW620 cells responded similarly in cell viability and colony formation assays, whereas CCD841 non-transformed colonic epithelial cells were resistant. For example, IC_50_ values for Aspalathin were in the range 20–31 μM in SW620 and SW480 cells vs. >120 μM in CCD841 cells (Supplementary Table S2). These findings implied the preferential targeting of colon cancer vs. normal colonic epithelial cells by the test agents. Immunoblotting of whole cell lysates revealed that with reduced BRD9 expression, test agents produced a marked loss of oncogenic c-Myc protein, induction of pRPA32 and pH2AX DNA damage/repair markers (Figure 4F, Supplementary Figure S1), and increased cleaved PARP and caspase-3 indicative of apoptosis (Figure 4F). The latter experiments implicated quercetin as the most effective polyphenol for apoptosis induction, although this was not the case in FACS-based assays (Figure 4E). This apparent discrepancy can be due to differences between the appearance of a cell surface apoptosis marker versus the cleavage of a nuclear enzyme by caspases during apoptosis, that might be resolved by additional time-course studies.

## 4. Discussion

BRD-containing proteins serve as scaffolds, transcription factors, and chromatin remodeling agents with critical roles in physiology and pathophysiology [2]. The interaction of BRD9 with SWI/SNF complexes represents an area of much current interest for cancer treatment [30,31,32]. BRD9 is overexpressed in many malignancies, including CRC (Figure 1) and Gastrointestinal Stromal Tumors (GISTs) [33]. We observed that siRNA-mediated loss of BRD9 expression in human colon cancer cells produced marked changes in phenotypic and molecular readouts (Figure 2), consistent with the overarching goal of targeting oncogenic functions arising from BRD9 overexpression.

Previous studies demonstrated that several residues in the ZA and ZB loops of the BRD9 bromodomain, such as Asp144, Ile53, Lys91, Thr104, Pro82, Asn140, Asn100, and Phe44, are important for optimal BRD9 inhibition [34]. Based on this information, natural compounds were screened via molecular docking in silico, which identified favorable interactions for natural polyphenols such as Epigallocatechin-3-gallate (EGCG), Equol, quercetin, and Aspalathin with the bromodomain of BRD9 (Figure 3A,B), and corroborated via BROMOscan^®^ and ITC experiments. Notably, an apparent Kd of 20 μM (Figure 3C) and IC_50_ values of 20–30 μM (Supplementary Table S2) fall within the physiological range for polyphenols and their bioactive metabolites [35,36].

Natural compounds with BRD9-targeting capability (Figure 3) had enhanced DNA damage/repair and apoptosis endpoints in cell-based assays, comparable to iBRD9 (Figure 4). Natural polyphenols lowered BRD9 protein expression in the relative order Equol > Aspalathin > Quercetin > EGCG (Figure 4F). The imperfect association between loss of BRD9 and c-Myc expression implicated additional factors in downregulating the oncoprotein in colon cancer cells. Multiple BRD9-binding sites reside within *MYC* enhancer elements [33,37], with various multiprotein complexes differentially regulating apoptosis outcomes in CRC and other malignancies [33,38,39,40]. Similarly, the lack of a good association between BRD9 expression and the phenotypic markers (Figure 4) can be attributed to additional effects of polyphenols on other molecular targets and signaling pathways that regulate antioxidant, anti-inflammatory, anti-proliferative, and cell cycle inhibitory actions [41]. Nonetheless, BRD9 expression and pRPA32 were interrelated (Figure 4F), and up to a 6-fold increase in pH2AX and pRPA32 expression in colon cancer cells treated with iBRD9 or natural polyphenols (Supplementary Figure S1) suggests that DNA damage/repair pathways are worthy of further investigation. This would include BRD9 knockdown and recovery experiments to discern the precise mechanisms.

Polyphenols occur in many foods and beverages, including green tea, curry spices, grapes, soy, and berries. They hold considerable promise for cancer treatment [42], including the bioactive metabolites [43], due to the targeting of epigenetic mechanisms, DNA damage responses and oncogenic signaling networks in cancer vs. normal cells [11]. Dietary polyphenols such as green tea-derived EGCG are known to activate cell cycle arrest and apoptosis in cancer cells [42,44]. Aspalathin from Rooibos tea also exerted anti-proliferative and proapoptotic effects in liver and colon cancer cells [45,46]. Quercetin from citrus fruits exhibited anticancer activity in colon cancer models [47] and enhanced the actions of BET inhibitors [48]. Equol is a microbial metabolite derived from the soy isoflavone daidzein, with anticancer activity in the breast and prostate [49]. Repurposing of natural agents and their metabolites as lead compounds for new BRD9-targeting antagonists would provide an avenue for future intervention in the clinical setting, and a strong rationale to continue investigation into dietary strategies for CRC prevention.

## 5. Conclusions

BRD9 overexpression in CRC is synonymous with oncogenic activity and provides an avenue for precision medicine. In human colon cancer cells, knockdown of BRD9, pharmacologic inhibition with iBRD9, and targeting of BRD9 overexpression via natural polyphenols resulted in marked changes in phenotype and molecular readouts, whereas normal colonic epithelial cells were resistant. Future studies will repurpose other nutrients as lead compounds for improved BRD9 inhibition and antitumor activity.

## Data Availability

See text for online sources and accessibility.

## Supplementary Materials

The following supporting information can be downloaded at: https://www.mdpi.com/article/10.3390/nu14204317/s1. Table S1: Bromodomain inhibition. Bromoscan™ screening at 10 µM of test agent; – not determined; Table S2: Concentration for 50% inhibition (IC50) values in colon cancer (SW620, SW480) and normal colonic epithelial cells (CCD841) after 72 h of treatment, based on cell viability and colony formation assays as in Figure 4A,B; Figure S1: Densitometry data for (A) pH2AX and (B) pRPA32 and (C) calculated fold change compared to vehicle, from the immunoblotting of SW620 cell lysates shown in Fig 4F.
