# Supplementary material for: BRD9 Inhibition by Natural Polyphenols Targets DNA Damage/Repair and Apoptosis in Human Colon Cancer Cells
Source: Nutrients. 2022 Oct 15;14(20):4317. doi: 10.3390/nu14204317 (PMC9610492; doi:10.3390/nu14204317)
Supplement: Supplementary file 1 [file nutrients-14-04317-s001.zip › Kapoor et al Supplementary Figs.pptx]

## Slide 1
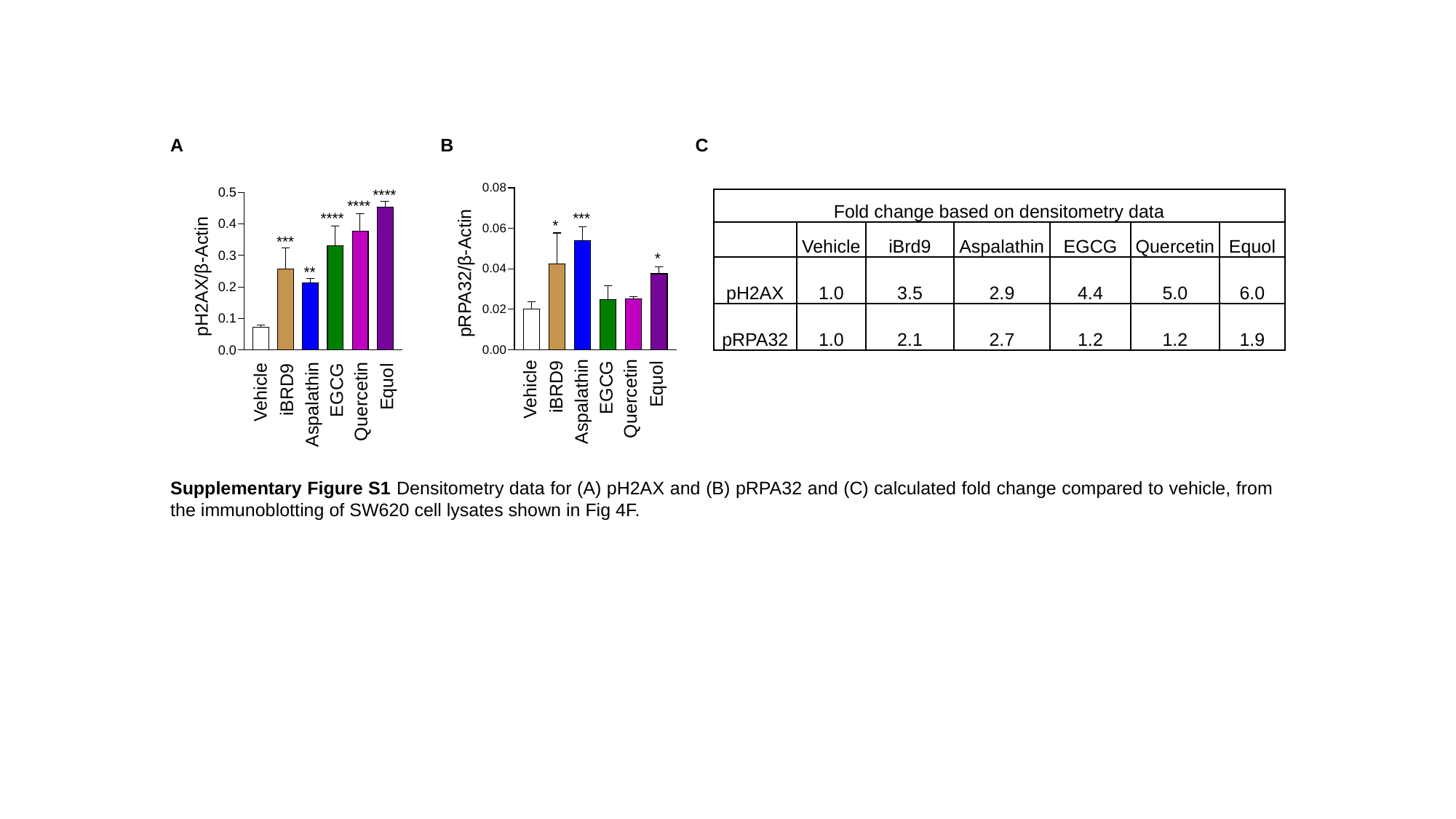

A
B
C
****
| Fold change based on densitometry data | | | | | | |
| --- | --- | --- | --- | --- | --- | --- |
| | Vehicle | iBrd9 | Aspalathin | EGCG | Quercetin | Equol |
| pH2AX | 1.0 | 3.5 | 2.9 | 4.4 | 5.0 | 6.0 |
| pRPA32 | 1.0 | 2.1 | 2.7 | 1.2 | 1.2 | 1.9 |
****
****
***
*
***
*
pRPA32/β-Actin
**
pH2AX/β-Actin
Equol
iBRD9
Equol
EGCG
Vehicle
iBRD9
EGCG
Vehicle
Quercetin
Aspalathin
Quercetin
Aspalathin
Supplementary Figure S1 Densitometry data for (A) pH2AX and (B) pRPA32 and (C) calculated fold change compared to vehicle, from the immunoblotting of SW620 cell lysates shown in Fig 4F.

## Slide 2
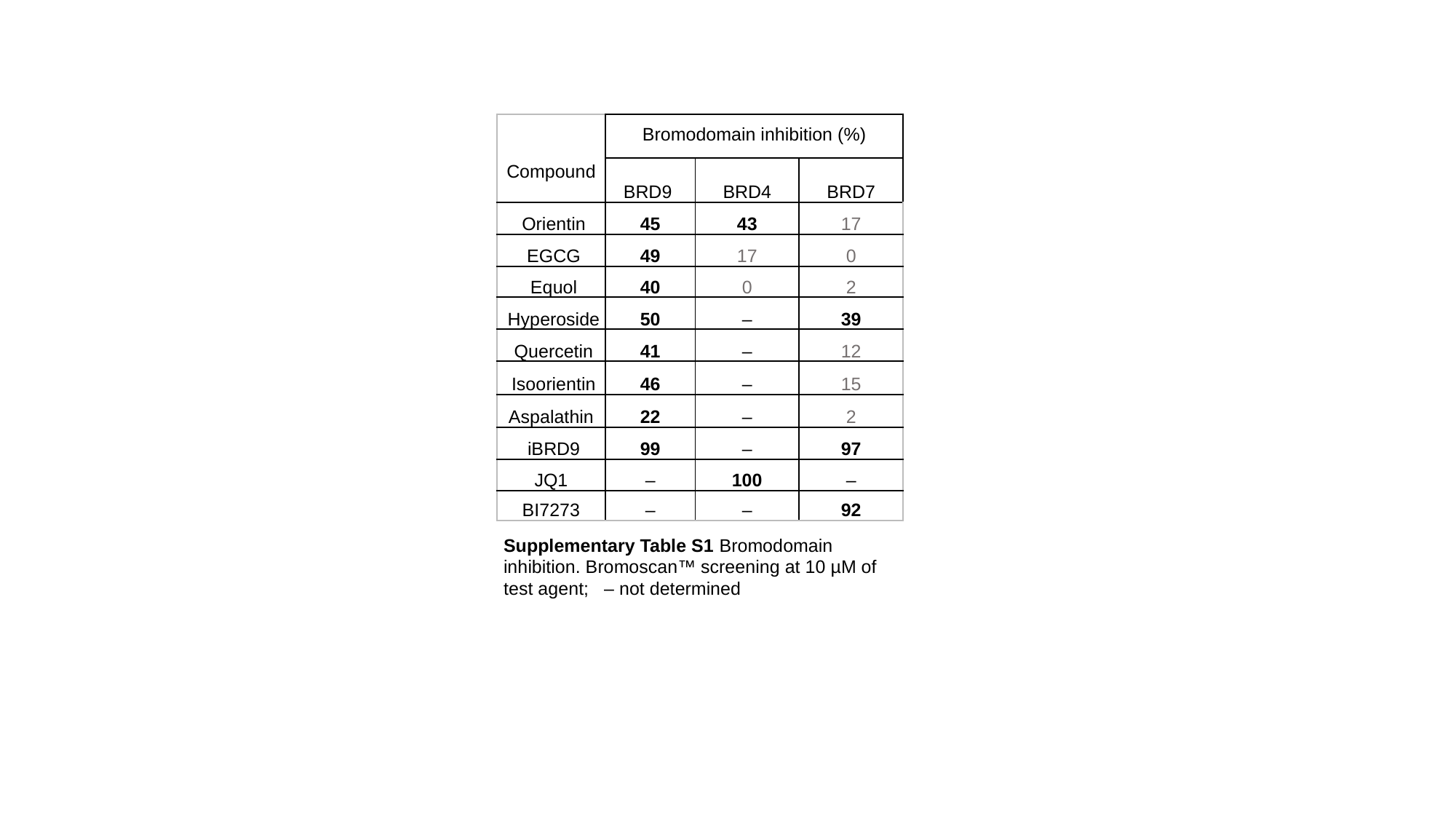

| Compound | Bromodomain inhibition (%) | | |
| --- | --- | --- | --- |
| Compound | BRD9 | BRD4 | BRD7 |
| Orientin | 45 | 43 | 17 |
| EGCG | 49 | 17 | 0 |
| Equol | 40 | 0 | 2 |
| Hyperoside | 50 | – | 39 |
| Quercetin | 41 | – | 12 |
| Isoorientin | 46 | – | 15 |
| Aspalathin | 22 | – | 2 |
| iBRD9 | 99 | – | 97 |
| JQ1 | – | 100 | – |
| BI7273 | – | – | 92 |
Supplementary Table S1 Bromodomain inhibition. Bromoscan™ screening at 10 µM of test agent; – not determined

## Slide 3
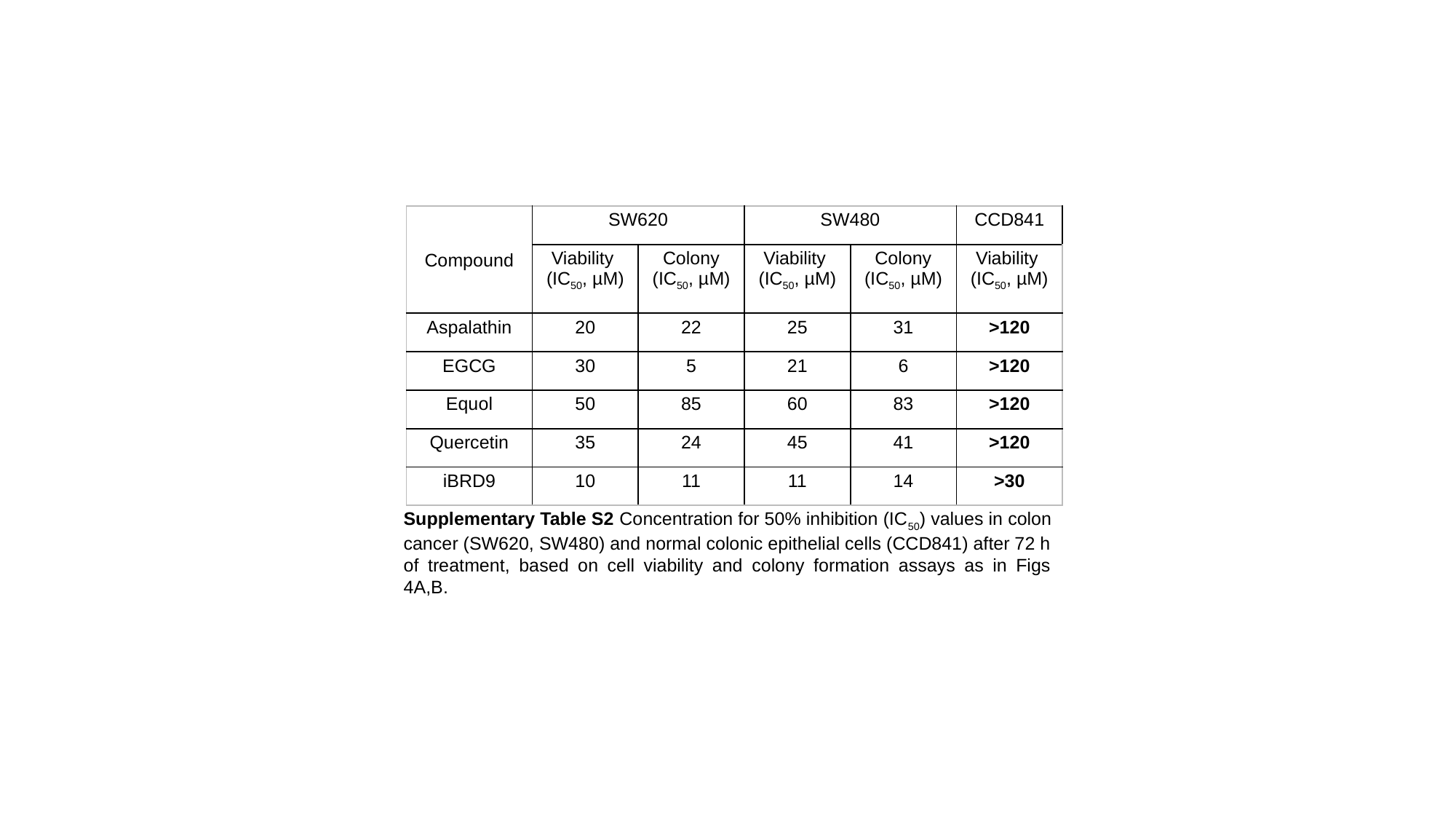

| Compound | SW620 | | SW480 | | CCD841 |
| --- | --- | --- | --- | --- | --- |
| | Viability (IC50, µM) | Colony (IC50, µM) | Viability (IC50, µM) | Colony (IC50, µM) | Viability (IC50, µM) |
| Aspalathin | 20 | 22 | 25 | 31 | >120 |
| EGCG | 30 | 5 | 21 | 6 | >120 |
| Equol | 50 | 85 | 60 | 83 | >120 |
| Quercetin | 35 | 24 | 45 | 41 | >120 |
| iBRD9 | 10 | 11 | 11 | 14 | >30 |
Supplementary Table S2 Concentration for 50% inhibition (IC50) values in colon cancer (SW620, SW480) and normal colonic epithelial cells (CCD841) after 72 h of treatment, based on cell viability and colony formation assays as in Figs 4A,B.
